# Gaining stakeholder perspectives to shape a produce prescription program to improve maternal and birth outcomes: a qualitative study

**DOI:** 10.3389/fpubh.2024.1462908

**Published:** 2025-01-15

**Authors:** Helene Vilme, Fang Fang Zhang, Perrie O’Tierney-Ginn, Chenchen H. Sun, Oyedolapo A. Anyanwu, Rukhshan Fahmi, Sara C. Folta

**Affiliations:** ^1^Friedman School of Nutrition Science and Policy, Tufts University, Boston, MA, United States; ^2^School of Medicine, Tufts University, Boston, MA, United States

**Keywords:** produce prescription, maternal health, nutrition, food insecurity, food is medicine

## Abstract

**Introduction:**

Nutrition during pregnancy significantly impacts maternal and birth outcomes. A key factor contributing to the rise in adverse maternal and birth outcomes is poor nutrition. Produce prescription programs have the potential to address pregnancy-related adverse outcomes such as hypertensive disorders and gestational diabetes, but scientific evidence is limited.

**Purpose:**

To conduct qualitative interviews to gain an in-depth understanding of how, why, and in what context should produce prescriptions be implemented to best meet the needs of pregnant women in a clinical setting.

**Methods:**

We conducted interviews with 11 patients with low incomes and/or experiencing food insecurity and 11 clinic staff from a major metropolitan OB/GYN clinic. Interview questions were designed to understand attitudes toward participating in or helping implement a produce prescription program. We analyzed the data using a deductive qualitative content analysis approach.

**Results:**

Both patients and clinic staff perceived many benefits to this type of program, including easing financial strain, removing barriers to access, and addressing nutrition security during pregnancy. Both groups described a need to consider participants’ autonomy in the program design. Patients also perceived some drawbacks to the home delivery aspect, such as limited participation by patients due to unstable housing. Staff expressed some concerns about the staff time needed to implement this type of program.

**Conclusion:**

There was strong support for produce prescription programs for this population; however, results indicate that they may best meet needs if patient autonomy and delivery-related barriers are considered in the design. Designating screening and enrollment tasks for ancillary staff may facilitate implementation in clinics.

## 1 Introduction

Over the past three decades, in the United States, the prevalence of hypertensive disorders in pregnancy has increased by 149%; in the same period, the prevalence of gestational diabetes has increased by 261% (1). These prevalence rates show a disparate difference among women from racial and ethnic minority groups and those from socioeconomically disadvantaged groups (2, 3). Maternal nutritional status is a strong predictor of pregnancy complications (4, 5) and plays a central role in birthing outcomes (6). Furthermore, studies have shown that women in the United States have inadequate intake of nutrients during pregnancy (6, 7). Drivers of inadequate nutrient intake or poor nutrition among pregnant women are multifaceted and include a lack of access to nutritious and healthy foods (7, 8). Specifically related to pregnancy, research indicates that 6% of pregnant women experience food insecurity (9).

A growing body of research suggests that Food is Medicine (FIM) interventions—medically tailored meals, medically tailored groceries, and produce prescriptions administered through the healthcare system—are cost-effective and improve household food security while also addressing health disparities (10). Among the most promising FIMs, produce prescriptions, which involve clinicians providing guidance and assistance for patients to access healthy produce, either through financial incentives or direct provision of produce, have demonstrated effectiveness in increasing the consumption of fruits and vegetables and improving clinical outcomes, including reduced hemoglobin A1c (HbA1c), blood pressure, and body mass index, among patients with diabetes, hypertension, or obesity (11, 12). However, the evidence for the impact of produce prescription interventions on maternal and birth outcomes is still limited (13, 14).

To ensure that the full potential of a produce prescription program for pregnant women is reached, it is essential to obtain input from key stakeholders to assess how, why, and in what context produce prescriptions will best meet their needs. Therefore, the purpose of this study was to conduct interviews with patient and clinical staff stakeholders to gain the information needed for effective design and delivery of produce prescriptions intervention for improving maternal and birth outcomes and reducing disparities.

## 2 Materials and methods

### 2.1 Study design and participants

The study team initially received internal university funding to develop and pilot a produce prescription program for pregnant women. Before commencing with the pilot study, the team obtained additional funding and conducted the formative research described in this manuscript. Specifically, we conducted in-depth interviews with pregnant women and clinical staff from a major metropolitan Obstetrics and Gynecology (OB/GYN) clinic from September 2022 through April 2023. Patients were identified by the OB/GYN social worker of the clinic. The social worker informed patients about the study and asked permission to share their contact information with the research team. The patients who agreed to be contacted were screened by phone by research staff. Inclusion criteria were as follows: (1) age 18 years or older; (2) at least one of the following: economically disadvantaged (household gross income ≤130% of the Federal Poverty Level); a racial or ethnic minority (African American, American Indian and Alaska Native, Asians and Pacific Islander, and Hispanic); having Medicaid or no health insurance; food insecure using a 2-item screener (15); and (3) willing to be recorded.

Clinic staff who would have a role in a produce prescription program by enrolling patients, issuing the prescription, tracking incentives, and/or implementing a nutrition education program were recruited via an email from or identified by one of the research team members. Staff included physicians, residents, and a nurse navigator, a physician assistant, and a practice coordinator.

### 2.2 Interview procedures

A protocol and semi-structured interview guides were developed following best practices for the qualitative work (16). The guide for potential program participants included questions about overall interest in and thoughts about the produce prescriptions, reasons for interest or non-interest, additional information needed for decision-making, perceived barriers to participation, program design that would hinder or facilitate participation (e.g., amount and types of produce; content and format of the nutrition education component). The guide for clinic staff was based on the Consolidated Framework for Implementation Research (CFIR) (17, 18) and included questions about the perceived need for the program, program fit with usual clinic practices, barriers to and facilitators of implementation, and self-efficacy for and willingness to implement the program.

Trained research team members scheduled and conducted in-depth interviews via zoom and in-person. The duration of the interviews was approximately 30 min for clinic staff and 60 min for patients. All discussions were audio-video recorded and transcribed. Transcripts were labeled with the unique study IDs to link the subjects to the identifiers. Patients received $25, and clinic staff received $50 as remuneration for their participation. We originally designed all interviews to be 60 min and planned to provide all participants (clinic staff and patients) with $25 remuneration. However, after failing to recruit clinic staff, we conferred with our collaborators, who suggested decreasing the duration of the interviews and increasing the remuneration amount to improve the recruitment of these busy professionals. We were successful at recruitment after making these changes.

### 2.3 Data analysis

Audio recordings were transcribed and then coded using NVivo software (V.12, QSR International, Doncaster, Australia). We used a directed qualitative content analysis approach (19), which is fundamentally deductive, to analyze the data. Initial codebooks for patient and clinic staff interviews were drafted based on the interview guides and then revised slightly based on a review of the transcripts. Inter-rater reliability was established by independent coding of one patient transcript and one clinic staff transcript. A kappa coefficient of 0.8 or greater at each code was deemed as acceptable (20). We discovered minor differences in interpretation at less than 10% of codes and clarified code definitions. Themes were then determined based on the similarity of responses across transcripts. Finally, we examined patient and clinic staff results for areas of overlap and integrated the two sets of findings into the major topic areas.

## 3 Results

### 3.1 Socio-demographic characteristics

A total of 11 pregnant women and 11 clinical staff participated in the study. Among the patient sample, the median age was 24 years; seven had children, eight were Black/African American, and 10 experienced food insecurity (Table 1). Among the clinic staff, the median age was 33 years and the majority were female (Table 2).

**Table 1:** Patient characteristics (*n* = 11).

Age in years, median (min–max)	24 (18–37)
Having children in the household, *n*	
Yes	7
No	4
Race, *n*	
White	2
Black/African American	8
Native Hawaiian/Other Pacific Islander	1
Hispanic, *n*	4
Within 130% of the federal poverty level, *n*	9
Food insecure, *n*	10
Employment status, *n*	
Employed full time	2
Employed part time	2
Out of work <1 year	4
Out of work ≥1 year	3
Highest level of education, *n*	
College graduate	1
Some college	1
High school or GED	7
Some high school	1
Elementary	1
Born in the USA, *n*	11
Marital status, *n*	
Divorced	1
Never married	9
Member of unmarried couple	1

**Table 2:** Clinic staff characteristics (*n* = 11).

Age in years, median (min–max)	33 (25–57)
Gender, female	11
Race, *n*	
White	8
Asian	3
Hispanic, *n*	1
Title, *n*	
Nurse navigator	1
Physician	3
Physician assistant	1
Practice coordinator	1
Resident	5

### 3.2 Overview of themes

The results are presented by the major topics within the interview guide and the corresponding themes that emerged: (1) overall interest in the produce prescription program; (2) design; (3) barriers to and facilitators of participation; and (4) barriers to and facilitators of implementation.

### 3.3 Theme 1: overall interest in the produce prescription program

Patients and clinic staff had a keen interest in the produce prescription program and perceived a strong need for and many benefits of the produce prescription program. The patient participants explained that their interest in the program was due to the opportunity to ease some financial constraints. Another reason for their interest was that the produce would be delivered to their homes, so they would not have to worry about going to the store.

*It would help out like with budgeting, honestly, because fruits and vegetables are starting to get very expensive. So it would help out with budgeting. (Patient, age 18, no other children in household)*

*I feel like that is something that in a grocery store people are less likely to go to because produce can be a little bit more expensive sometimes. Getting it fresh produce delivered weekly and it’s free, like the program just sounds so good. All you have to do is just comply and work and cooperate. It just sounds too easy. It sounds way too easy to not do it. (Patient, age 20, two other children in household)*

*The program would help save a lot of time taking a trip to the grocery store, it gets delivered. (Patient, age 28, three other children in household)*

Similarly, clinic staff expressed interest in the program because it would meet a great need for the patients, set guidelines for what is healthy to eat, and provide easy access to healthy items. Additionally, clinic staff confirmed the importance of the produce serving as an opportunity to improve patients’ health outcomes.

*Yes, I think this is a good way to set a guideline of what is healthy to eat and then have them have access to that in an easier way. (Clinic staff, practice coordinator, age 25).*

*I think that having something like this, especially at a point in someone’s life or healthcare journey where they are not only having to nurture themselves but also their fetus is particularly important. I think that after seeing so many patients with diabetes and the healthcare outcomes that are related to that, I think that having a program like this can be a very, like, good upstream way of targeting the root cause of a problem. (Clinic staff, resident, age 29)*

In addition, the clinic staff highlighted the importance of food-related programs that focus on food security, particularly for sub-populations that may have challenges obtaining and affording nutritious foods, especially while pregnant. Furthermore, clinic staff suggested that implementing programs like produce prescriptions might address transportation barriers through delivery.

*I think that it would be very, very helpful. We see a lot of patients with food insecurity and diabetes or other reasons to be a-- pregnancy, obviously, wanting to have a more nutritious diet, but it’s hard I think to get access to it. Obviously, cost is a factor. Convenience is a factor, transportation. A lot of our patients do not have any reliable means of transportation. I think this would be a really excellent thing for our patients. (Clinic staff, resident, age 33)*

### 3.4 Theme 2: program design

Patients and staff shared their thoughts about the logistics of program implementation and the content and format of the nutrition education aspect.

#### 3.4.1 Logistics of the program

*Mode of delivery, duration, preferred fruits and vegetables, and quantity* emerged as sub-themes. Patients were asked about each of these aspects. As the focus of the clinic staff interviews was on implementation, they were not asked about program logistics; however, the mode of delivery came up in some of the interviews. The intended plan was to deliver a box of fresh produce weekly to patients’ homes. The patients stated that they would have no issue with the weekly delivery; however, some patients and clinic staff raised the concern that in situations where the housing is not stable or secure, it might be better to arrange for a pick-up venue. Furthermore, patients recommended that there should be some notice provided to the recipients prior to the delivery date.

*I do not think I’d run into any problems. Right now, I’m staying with my mom and she’s a homeowner, so it’s not like as if I’m in an apartment still where I would have the potential issue of people taking or stealing my deliveries. (Patient, age 24, one other child in the home)*

*Okay. If you are busy with work until later, someone might not always be home all the time to pick up the groceries. Are they calling people when they are coming so somebody knows, “Oh, this person’s coming to my home.” If you thought to give somebody a week that’s fine. I’m sure they’ll be able to find somebody to pick up their fruits and vegetables. They’ll be home for that. (Patient, age 37, three other children in the home)*

*It will be need to be delivered. I think that sometimes that can be challenging for patients because they may or may not have stable housing and maybe staying at a friend's house or a family member's place. It'll be necessary to confirm a sustainable address, which may change over the course of the pregnancy. (Clinic staff, age 32)*

One patient stressed the need to ascertain that people really want the produce by making the delivery option to be by pick-up rather than drop-off in people’s homes.

*I think that people who really want it would actually go to pick it up because that would show that they actually want it and will eat it. I think it might just go to waste if it’s delivered because they might just give it away or just let it sit there or will not go to use. (Patient, age 30, two other children in the home)*

Clinic staff expressed support for home delivery because of the opportunity to improve access to food, especially among patients who are busy taking care of family members.

*Honestly, I think the delivery aspect of it is really important. I know that there are some ways for patients to get food that’s subsidized, but it still requires them to go somewhere to pick it up. I think having the delivery aspect of it gives even better access to the food. (Clinic staff, resident, age 31)*

In terms of *duration* of a produce prescription program, patients were asked about the proposed program duration of 16 weeks. Most patients felt that this was an adequate time to allow people to develop some form of habit around eating fruits and vegetables and to help those for whom affordability might be an issue. A few expressed that it could be longer, but that 4 months of free produce was better than nothing. One patient suggested doing a trial run for a shorter period in order to gauge interest before doing it for 4 months.

*I feel like incorporating it for four months, incorporating fruits and veggies into your diet and getting comfortable with it, I feel like after that time period of four months, it’s very easy to just stick with it. Because your body will start to crave it and you know what to do with it now. I feel like it’s really easy to make it a habit after that four months. (Patient, age 20, two other children in the home)*

*I would expect a little bit longer just because for me being pregnant, and then after being pregnant, nursing the kid, I would want my child also to grow up carrying on that same diet. It’s nice if I’ll be able to maintain it while I’m pregnant, but I would also want it to be retained for my child as well. If it could be longer, I feel like that would be amazing, but I do not believe it’s too short. (Patient, age 24, one other child in the home)*

*I think they should start with something smaller, maybe two months, and test it out first to see how many people would probably stick with it and if it’s getting used. Then touch base with the people again after the two months, and then decide if they should do it longer. I think start smaller. (Patient, age 30, two other children in the home)*

Concerning the *quantity of fruits and vegetables*, patients were asked about the adequate amount, and they were also asked to describe issues that could potentially arise with getting too little or too much produce. Patients explained that it is hard to quantify the amount of fruits and vegetables they would consider appropriate because it would depend on many factors, such as the size of the household, whether the household has facilities to preserve produce, such as a refrigerator or freezer, and how much each individual likes to consume. They felt that getting more than adequate may likely lead to food waste, while receiving less than adequate should pose no additional problem since the produce is free.

*I think if you are getting it every week, they should give a supply of what you would normally use throughout the week, and nothing more or less, so you can have a fruit and vegetable for each meal for the seven days. (Patient, age 24, no other children in the home)*

*I think if they got too much, it would just go to waste and that’s less people. I think the smaller amount that’s being delivered is more likely to be used. (Patient, age 30, two other children in the home)*

*I do not think there would really be any problems, honestly if they did not get enough or not. (Patient, age 18, no other children in the home)*

One participant was concerned that not getting enough could make them resort to less healthy food options:

*That I feel like it would resort to wanting to find alternative, like for sweets. Again, me, it’s a bit expensive where I am to buy fruits, so if I do not have enough money to buy it, it’s cheaper to buy sweets and all these pastries and fatty foods. That can lead someone to want to eat bad because junk food is more inexpensive than fruits. (Patient, age 24, one other child in the home)*

#### 3.4.2 Content and format of the nutrition education component

Among the patients and clinic staff, interview questions on the nutrition education component of the produce prescription addressed topics related to the format and content. Thus, the c*ombination approach* emerged as a sub-theme. Some of the patient participants indicated that they wanted to receive printed materials, while others preferred electronic delivery formats such as email or text messages or some form of app delivery.

*Oh, probably an email. Do not waste paper. People probably just throw it away. (Patient, age 30, two other children in the household)*

*I think the newsletter would probably be better. I think emails get lost, at least for me, or I see it and then I neglect to go back to it and look at it, but a newsletter’s always kicking around. (Patient, age 30, two other children in the home)*

*I do not know if you guys have like a little app or something, like, “Today eat a banana or tomorrow.” A little app or something. You know how they have the app for the baby and how they grow, like the same thing. (Patient, age 22, no other children in the home)*

Clinic staff also suggested a *combination approach*; most recommended the use of electronic media and a few recommended printed materials and in-person counseling as the modes of delivery of the nutrition education component. They expressed concerns about language barriers as they provide care to patients from diverse linguistic backgrounds who may not be comfortable with English.

*Yes, the one thing that comes to mind is that so many of our patients don't speak English. I don't know if you were going to do paper what languages it would be available in? Sometimes a video that has very basic pictures and information can be helpful in populations who don't speak English. Same thing with pamphlets, I guess just pictures and just making sure it's in the languages that are appropriate. I think that's the biggest thing that we struggle with is when we have initiatives like this to make them truly accessible, they need to be translated….Okay. Videos or infographics that are translated into languages that the patients speak. (Clinic staff, resident, age 33)*

With respect to the frequency of receiving the nutrition education materials, patients reflected a preference for weekly or every other week distribution. One participant added the caveat that it should be of concise length.

*I feel like it’s very helpful to know little things like that. Getting it on a weekly basis, making it a habit like that will definitely make it stick much easier if you continue to do something like that. (Patient, age 20, two other children in the household)*

*I would say do not make it so overwhelming as far as it being definitely has to read. I’ll say make it something light, they have to read it every week. (Patient, age 37, three other children in the household)*

Regarding the content of the program, both patients and clinic staff suggested including simple healthy meal recipes and the nutritional value of types of foods and their implications on the human body, particularly as it relates to their pregnancy state. Of note, clinic staff stressed that differences in patients’ health status should be kept in mind while composing this information for the content.

*Probably what’s best for the baby. What different meals I can eat and things like that, and different preparations and how to prep my food to like not eat too much or too less. That’s something I would ask for. (Patient, age 22, no other children in the household)*

*I feel like the vegetables and the fruits and the things that they give, when we do eat these fruits and these vegetables, what do they do to our body? What are they helping us with? What specifically? I feel like a lot of people run to medicine, a lot of people run to the other things because they do not realize that the food you eat plays such a big part in how your body reacts. (Patient, age 20, two other children in the household)*

*A different recipe every week or something on the card with the vegetables, one of the vegetables that are in the box or something. I think that would be pretty cool. (Patient, age 24, no other children in the household).*

*I think during pregnancy, a lot of people are just told to keep eating how they have been and what their appropriate weight gain should be throughout their pregnancy but I don't think they ever get specific information about how many calories a day that means, and what types of foods it should be broken down into by, like a food pyramid. Which when you're pregnant you do need a little bit more calories per day but not a significant amount…I think that's what I would ideally focus on. Obviously, it's better to get nutrients and calories from fresh foods like that than packaged processed things. I think all of that should be explained, it would be helpful to be explained. (Clinic staff, physician assistant, age 30)*

Additionally, some clinic staff added that, alongside addressing the different health conditions of patients, the content should focus on the social aspects of the patients as well. Aspects like maternal guilt or the economic condition of the person or their family structures should be kept in mind prior to developing the content.

*I think the other really important component of nutrition education is that there is a lot of maternal guilt that goes around feeding yourself when you are pregnant… I think it's really important to keep in mind how we're presenting that information and not being like, "Well, if you do this it's really important for your baby." I think that that is a really hard framework for patients because they're all doing their best. I think increasing that maternal guilt is always a big risk of telling people what they should and shouldn't be eating. This is about a healthy pregnancy for you and so forming lifelong habits. Those are the things I focus on versus being like, "Feed your baby vegetables." [laughs] Then having them be like, "I only have money for pasta." It's hard. I've been a mom before so I remember being like, ‘Oh my God, what am I doing wrong? (Clinic staff, physician, age 38)*

*I think that something that would be really helpful would be, specifically for gestational diabetics, actual examples of healthy protein-filled snacks or meal choices. Sometimes we'll tell patients more like dogmas of avoid high-carbohydrate meals or avoid this category, but we don't actually give specific examples of like, "So a handful of almonds plus this," and specific examples for patients that are not just something that they have to Google but can actually adapt into their lives as pregnant patients will often need to eat more frequently and just have different needs than when they're not pregnant nutritionally….I think that working with a nutritionist ideally and having some realistic but good specific examples for patients that can be adapted for their family structure, not just for them personally, would also be a nice thing to incorporate, and again, get more patient buy-in. (Clinic staff, resident, age 32)*

### 3.5 Theme 3: facilitators and barriers to participation

Facilitators and barriers to participation were explored from the patient’s perspective. Sub-themes that emerged were *motivation and engagement strategies, perceived barriers to participation, and perceived downsides to the program*.

The motivation and engagement strategies theme captures the need to address both intrinsic (personal choice) and extrinsic (incentives) factors to increase participation in a produce prescription program. In particular, a patient indicated participation in a produce prescription program was a personal choice, denoting that autonomy is important. Furthermore, while produce prescription programs inherently assume participants are willing and motivated to consume fruits and vegetables, the patient highlights the role of external motivators beyond the consumption of fruits and vegetables, which aligns with the idea that tangible rewards or additional benefits may enhance engagement and adherence to the program. The specific quote is presented below.

*I think it’s probably a personal choice. I do not know if there’s anything that someone can do to make somebody eat more fruits. If you want to promote fruits and vegetables and get them to want to eat them more, totally offer a benefit other than the fruits and vegetables with them…Yes, like an incentive. If you guys participate in this or you eat the fruits and vegetables, then you’ll get something with it maybe. (Patient, age 30, two other children in the household)*

While many patients could not foresee any potential barriers to participating in a produce prescription program, some had issues with the mode of program delivery. For example, not having stable accommodation or even the timing of the delivery might be a barrier if the person was not at home when the produce was delivered and someone else took it. One participant was also concerned about not knowing the source of the produce.

*I feel like if someone does not have a stable place to live, it’d be very hard for them to participate in this program. I do not know. That’s about it. If you do not have a stable place to live to receive the box, I think that would be a very difficult way of [receiving it]. (Patient, age 24, no other children in the household)*

*Probably, it being delivered on my front doorstep. I do not think I would want that. I’m not home. It depends on the area. Someone might take it. You do not know where it’s been, if it’s dirty. It’s probably more better to go into the grocery store and purchase it yourself with a card or something. I would not—yeah, not knowing where it came from is going to be probably a concern for me, that it’s just dropped off. (Patient, age 30, two other children in the household)*

Most patients did not perceive any downside to the program. One participant did evoke the idea that the program is targeted at pregnant women whose physiological state might make them have some temporary aversion to certain foods. Another one felt that identifying some women as needy and sending them produce might be negatively conceived.

*Anything nutritional is there to help you, but … where there are some people that might feel like they are now considered someone that’s needy or someone that’s less fortunate. It could make an impact on someone, how they view themselves, but again, that’s just how the person views themselves. You can look at the glass either have full or half empty. Half full, meaning that it’s a program here to help you and half empty, where it’s like you are dependent on it, but they are still here to help you. It’s up to participants personally. (Patient, age 24, one other child in the home)*

### 3.6 Theme 4: facilitators and barriers to implementation

Among the clinic staff, the Consolidated Framework for Implementation Research (CFIR) was used to categorize reported facilitators and barriers within three of the five domains in congruence with the construct definitions for program implementation (see Appendix A). Specifically, facilitators within the *outer setting included patient needs and resources, and within the inner setting, a positive culture and compatibility* were important for the implementation of the program. Barriers within the outer setting were not reported. However, within the inner domain, *networks and communications* emerged as a potential barrier. Within the characteristics of the individual domain, *self-efficacy,* or the capability of the clinical staff to execute the courses of action to identify and refer patients to the program, emerged as a facilitator. Simultaneously, the *process and the engaging* constructs emerged as both facilitators and barriers.

Within the CFIR domain of the outer setting, the *patient needs and resources* construct emerged as a facilitator. Specifically, clinical staff expressed that if they were aware of such a resource (program) and that their patients would benefit from it, they would ask how to “make it happen” for their patients.

*I feel like with the right mode of communication to the patients and with the right support from the providers, it could be a really successful program. (Clinic staff, practice coordinator, age 25)*

Several constructs were explored within the inner setting CFIR domain, networks and communications emerged as a barrier while culture and compatibility materialized as facilitators. Pertaining to *networks and communications*, clinic staff indicated that transparent communication with the patient would be helpful and that in previous studies, the lack of communication impacted the program outcome within the clinic.

*I feel like with the right mode of communication to the patients and with the right support from the providers, it could be a really successful program. In other studies that we've seen the communication is just not there, the knowledge of the program is just not there, so they haven't been, I feel like, as effective as maybe the providers would like, but I feel like given the right resources and the right people, it would work. (Clinic staff, practice coordinator, age 25)*

Using the CFIR recommended use of the Competing Values Framework (CVF) (19), one of the four sub-codes related to the four dimensions of the CVF was team culture. A clinic staff stated that it would be a good fit for the clinic’s culture, asserting that they were very patient-oriented.

*I think it would be a good fit for our clinic's culture. We try to be very patient-oriented… not just about the medical needs of our patients, but also about all of the other things going on in their lives to the degree that we can. (Clinic staff, physician, age 38)*

Although the clinic staff indicated that the program would align with their clinic’s culture and were compatible with their clinic’s vision and mission, a barrier that emerged was *concern about the extra time* that might be required for these new responsibilities, especially screening and enrolling patients:

*I don't think it would affect our usual operations a lot, except maybe, like I said before, that initial prenatal visit, I think would have to be used to screen people. I think that would be the biggest thing it would affect. Those initial visits are an hour long so they are lengthy visits that are meant to cover all sorts of things like this. (Clinic staff, physician assistant, age 30)*

*It's just a question of time in terms of who discusses it with patients. If it's a quick thing where we can say, here's this program that we can offer you and they sign up and that's it, I think that would fit really well. (Clinic staff, resident, age 39)*

Clinic staff responses reflected *compatibility* as a facilitator within the domain of the inner setting. The clinic staff identified that the produce prescription program would be a tangible fit between the vision and mission of the clinics and the existing workflows. Moreover, the clinical staff shared that there were many areas of fit between their current responsibilities and the tasks the program intends to do in the future. Specifically, they indicated that they are accustomed to having patients of different ethnicities, backgrounds, different health needs, and connectedness to various food assistance programs. These comparisons gave them confidence about adopting the program in their clinical settings.

*I think that it ties in with being able to treat a patient as in recognizing that they are a whole person and not just limited to a person who's pregnant, who's sitting in front of me in clinic, but seeing them not only as a patient who has diabetes or high blood pressure or whatever it is… I think that there's a recognition of the systemic issues that contribute to those diseases and not just treating the illness but treating what caused or potentially contributed to the illness. (Clinic staff, resident, age 29)*

In terms of findings within the CFIR domain related to characteristics of individuals implementing the program, most of the clinic staff expressed confidence (*self-efficacy*) about identifying and referring patients to the program. They attributed it to their current system, which would make it comfortable for them to perform the task.

*I think that wouldn't be a problem. I think that most of us would feel pretty confident and comfortable offering this at least and saying, "Would you be interested in it?"….I think the biggest barrier to that is just people being transparent about what they really need and what would help them. I do think that women’s health is a good place for it. That's where people I think tend to be pretty open with us. We also do have a good set of social workers here who meet with a lot of our patients regularly and would probably be a big asset to this program if it were to go live. (Clinic staff, physician assistant, age 30)*

*I think good, as long as I have a validated survey that I can use to see if patients qualify because it's similar to when we screen people for postpartum depression…. I think having something that's validated would be really helpful. (Clinic staff, physician, age 40)*

Few were concerned about their ability to do the task. One added that for identification, it is important to have clarity in the screening questionnaire so important aspects are not missed.

*I wouldn't say I'm that confident because like I said, I don't see the patient in a clinical capacity. Their diet, their modes of eating and preparing food, that's something that should be discussed with somebody that knows their medical background. I feel like a lot of factors have to take place in understanding what to prescribe the patient and what goes into their nutrition choices. (Clinic staff, practice coordinator, age 25)*

*That's not something we routinely ask of patients. I'm curious how you would even determine that. I don't know what patient's income….I sometimes know something about their race, but to be honest with you, I'm probably assuming a lot of the time. I don't always know how they identify their race. That probably wouldn't work terribly well either. Our intake form doesn't ask about race in a very good way. It really is like a write-in and so I think we would probably be getting a lot of misses or a lot of wishy-washy answers. We don't ask about education level at all…. which is to say you just ask the patient. (Clinic staff, physician, age 38)*

Within the process domain of CFIR, *planning and engaging* emerged as facilitators and barriers. The clinic staff emphasized the need for a plan (i.e., methods and tasks) that includes easy implementation and clear communication processes, particularly regarding the understanding of the roles and responsibilities among the providers. Failure to have easy implementation and communication processes could lead to barriers to implementation. Regarding the engagement of potential key stakeholders (e.g., providers and staff), the clinic staff intimated that having training for the stakeholders may facilitate better implementation of the produce prescription program. Similarly, not having the necessary training could be a barrier to implementation.

*I think that the easier the enrollment and implementation process is, the more likely it is that we would use it. Then the other thing that sometimes happens when we implement really awesome things is that there's not enough training and there's not available sheet of paper to say, here are the steps to do it… Don't leave it up to their providers to decide how to do it. To say this is how it should be implemented. If this is what makes the patient enroll in as a candidate, here's how they enroll, done. (Clinic staff, resident, age 39)*

*Sometimes a face-to-face with the providers is helpful or having somebody at least in the clinic that knows exactly what the protocol is. We have Friday meetings every Friday. Somebody coming and being like, "Let me take two minutes of your time to show you how to fill this form out and what it means." That kind of thing can be helpful so that everyone-- not that everyone attends, but probably about half the providers do. That way you could reach the most people, I think. (Clinic staff, physician, age 38)*

## 4 Discussion

This study investigated the perspectives of patients and clinic staff at a major metropolitan Obstetrics and Gynecology (OB/GYN) clinic on the information required to effectively design and implement a Food is Medicine produce prescription program to improve maternal and birth outcomes and reduce disparities. Patients and clinic staff expressed interest in the program, underscoring the necessity and several benefits of a produce prescription program that would alleviate budgetary limitations, remove access hurdles, and address nutrition security during pregnancy. These findings are consistent with prior studies, in which participants in produce prescription programs reported that the benefits include removing barriers to accessing and purchasing produce (20). One of the largest evaluation studies of produce prescription programs, analyzing individual-level data in 22 sites across 12 US states from 2014 to 2020, lends support to the effectiveness of produce prescription programs at addressing access to fruits and vegetables: in that study, the odds of being food insecure decreased by one-third (12). Moreover, a study leveraging physician’s prescription to encourage healthy behavior change and reduce financial barriers to healthy eating (21). Furthermore, a scoping review assessing the logistics of food prescription programs discovered considerable gaps and discrepancies in the literature (22). Our study contributes to the literature by engaging stakeholders to understand logistical factors that will best serve their needs.

Regarding the nutrition education component, both patients and clinic staff proposed including easy healthy meal recipes as well as the nutritional value of types of foods and their effects on the human body, coinciding with elements of produce prescription programs in the literature (23, 24). It is worth noting that a finding that has yet to be explored in the design of prescription programs is the social aspect of developing nutrition education materials, such as maternal guilt and the economic condition of the person or their family structures. In previous research, the focus was simply on mom guilt and feeding outcomes rather than designing materials to counteract this feeling (25). In future studies, it will be critical to consider maternal guilt as it can influence how participants perceive and engage with produce prescription programs. By designing interventions that are supportive, empowering, and sensitive to this emotion, programs can foster a more positive and affirming experience for women and their families during this transformative time in their lives.

Both the patient and clinic staff discussed the facilitators and barriers. While patients focused on the barriers and facilitators to participation, clinic staff discussed the barriers and facilitators to implementing the produce prescription program. Patients reported that program benefits and incentives were important motivators for participation. Furthermore, motivation and engagement strategies of a produce prescription were discussed, capturing both intrinsic (personal choice) and extrinsic (incentives) factors to increase participation in a produce prescription program. While many patients did not anticipate any potential barriers to participating in a produce prescription program, some saw drawbacks to the home delivery aspect. In a Food is Medicine produce prescription program, Owens et al. noted that 20% of the participants were experiencing housing insecurity in addition to food insecurity, illustrating the influence of multiple social factors that may impact health outcomes (26).

To explore the clinic staff’s perspectives on the facilitators and barriers to the implementation of the produce prescription program, three of the five CFIR domains were used as initial coding categories, elucidating seven of the CFIR constructs. Research has underlined the necessity of considering patients’ needs and resource availability as underestimating these aspects will have an influence not only on implementation but also on program outcomes (27, 28). Furthermore, clinic staff noted that the produce prescription program would be a tangible fit between the clinic’s vision, mission, and existing workflows. The cohesion of the clinic’s vision and existing workflow emerged as facilitators and aligned well with the construct of compatibility within the domain of the inner setting. Research has also shown that aligning an workflow of an organization in a clinical context can lead to successful nutrition program implementation (28). However, a key barrier identified was the additional time required for screening and enrolling patients, a challenge directly addressed by Recommendation 17 from the Mainstreaming Produce Prescriptions: A Policy Strategy Report (29). The report underscores that while healthcare providers recognize the value of these activities, many feel ill-equipped and unsupported in integrating them into their routines. These findings reflect the need for technical assistance, funding, and staff training to mitigate resource constraints and improve preparedness as noted in the policy strategy report. A significant barrier is the additional time required for screening and enrolling patients. Moreover, the networks and communications construct of the inner setting of CFIR, or the nature and quality of the webs of social networks and the nature and quality of formal and informal communications within an organization, can serve as a facilitator or barrier to implementation. In this study, clinic staff noted that networks and communications could potentially be a barrier to program implementation due to a lack of transparent communications.

Additionally, the clinic staff perceived the culture of values, beliefs, and norms within the inner setting of the CFIR positively, emphasizing that the specific health needs and desired outcomes of individuals drive all provided care. Taher et al., using CFIR to map food security screening for the development of primary care practice guidelines, also noted the importance of culture of the clinic, with clinicians stating, “We do things differently here,” and “the way we do it is we want to reach everyone,” denoting a universal food distribution process to reduce the stigma of food insecurity (28). In terms of the characteristics of individuals implementing the program, most clinic staff reported confidence in identifying and recommending patients to the program. Self-efficacy, or confidence in program implementation, has been reported to be a major facilitator when present and a barrier when absent (30). Regarding the CFIR process domain, clinic staff noted that planning and engaging constructs emerged as facilitators and barriers to program implementation. While program planning and stakeholder engagement methods may vary across settings, they are commonly viewed as strategies to enhance effective implementation by developing the local capacity to utilize the intervention at both group and individual levels (31). As such, creating a course of action for the program and engaging the right stakeholders are critical to the success of the prescription program.

### 4.1 Strengths and limitations

There are several strengths of this study. First, this study contributes valuable insights by expanding the limited information available for designing and implementing produce prescription programs tailored to pregnant women. Second, this study uses CFIR, which enables the assessment of contextual capacity and needs, by providing defined constructs that identify potential barriers and facilitators to implementing a produce prescription program in specific settings. Despite its strengths, it is crucial to acknowledge that the sampling was geographically limited, which may limit the applicability of the findings to pregnant women from different regions of the United States. It would be beneficial for future research to include a more diverse geographic sample to ensure broader implications for prescription programs tailored to pregnant women. Additionally, the clinic staff provided valuable perspectives on the feasibility and challenges of implementing a produce prescription program in the clinical setting, particularly in terms of logistical considerations such as produce delivery methods and patient engagement strategies. However, future studies could benefit from including a larger sample of clinical staff from various types of healthcare settings to explore how these factors might differ across diverse practice environments. Furthermore, several critical aspects to address when considering the successful implementation of a produce prescription program are produce delivery logistics, partnerships with farmers or vendors, payment mechanisms, insurance requirements, and contingency plans for delivery issues represent significant operational challenges for produce prescription programs. In our study, we did not specifically inquire about these aspects during interviews with clinic staff or patients. We believe these subjects may be outside the scope of their expertise, and their responses did not naturally address these factors as barriers or facilitators to the program. A future study to explore these aspects in detail would contribute to the literature.

## 5 Conclusion

This study provides insights into the elements needed for designing and delivering a produce prescription program to improve maternal and birth outcomes. Furthermore, this study sheds light on the challenges and opportunities that may arise during program implementation. Overall, both patients and clinic staff reported great interest in and support for produce prescription programs. We discovered that three of the five CFIR domains helped identify possible barriers and facilitators to the produce prescription program. For example, the construct of culture and the compatibility of the inner setting emerge as facilitators, indicating a positive organizational culture that is patient-oriented and has a vision and goal that coincide with addressing social factors that influence health. The network communication construct of the outer setting domain and the planning and engagement constructs of the process domain were viewed as both barriers and facilitators by clinic staff. Overall, the findings of the study may facilitate the design and implementation of a program aimed at eliminating maternal nutrition disparities.

## Data Availability

The datasets presented in this article are not readily available because original qualitative datasets will not be shared. Requests to access the datasets should be directed to Sara.Folta@Tufts.edu.
