# Supplementary material for: Gaining stakeholder perspectives to shape a produce prescription program to improve maternal and birth outcomes: a qualitative study
Source: Front Public Health. 2025 Jan 15;12:1462908. doi: 10.3389/fpubh.2024.1462908 (PMC11774915; doi:10.3389/fpubh.2024.1462908)
Supplement: Supplementary file 1 [file Table_1.DOCX]

Supplementary Material

# Supplementary Table

| **Domain** | **Construct** | **Description** | **Clinic Staff input summary and quotes** |
| --- | --- | --- | --- |
| **Outer setting** | | | |
|  | Patient needs and resources | The extent to which patient needs, as well as barriers and facilitators to meet those needs, are accurately known and prioritized by the organization. | **Facilitator**  Clinical staff expressed that if they were aware of such a resource (program) and that their patients would benefit from it, they would ask how to “make it happen” for their patients. |
| **Inner setting** | | | |
|  | Networks and communications | The nature and quality of webs of social networks and the nature and quality of formal and informal communications within an organization. | **Barrier**  Clinic staff indicated that transparent communication with the patient would be helpful and that in previous studies, the lack of communication impacted the program outcome within the clinic. |
|  | Culture | Norms, values, and basic assumptions of a given organization. | **Facilitator and barrier**  Clinic staff stated that it would be a good fit for the clinic’s culture, asserting that they were very patient-oriented. However, extra time for completing the responsibilities was a concern. |
|  | Compatibility | The degree of tangible fit between meaning and values attached to the intervention by involved individuals, how those align with individuals’ own norms, values, and perceived risks and needs, and how the intervention fits with existing workflows and systems. | **Facilitator**  Most of the clinic staff expressed confidence (self-efficacy) about identifying and referring patients to the program. They attributed it to their current system, which would make it comfortable for them to perform the task. |
| **Characteristics of Individuals** | | | |
|  | Self-efficacy | Individual belief in their own capabilities to execute courses of action to achieve implementation goals. | **Facilitator and barrier**  Most of the clinic staff expressed confidence (self-efficacy) about identifying and referring patients to the program. They attributed it to their current system, which would make it comfortable for them to perform the task. On the hand, some were concerned about their ability to do the task. |
| **Process** | | | |
|  | Planning | The degree to which a scheme or method of behavior and tasks for implementing an intervention are developed in advance, and the quality of those schemes or methods. | **Facilitator and barrier**  The clinic staff emphasized the need for a plan (i.e. methods and tasks) that include easy implementation and clear communication processes, particularly regarding the understanding of the roles and responsibilities among the providers. Failure to have easy implementation and communication processes could lead to barriers to implementation. |
|  | Engaging | Attracting and involving appropriate individuals in the implementation and use of the intervention through a combined strategy of social marketing, education, role modeling, training, and other similar activities. | **Facilitator and barrier**  Regarding the engagement of potential key stakeholders (e.g., providers and staff), the clinic staff intimated that having training for the stakeholders may facilitate better implementation of the produce prescription program. Likewise, not having the necessary training could be a barrier to implementation. |

**Appendix A:** Consolidated Framework for Implementation Research constructs--clinic staff input on the barriers and facilitators to implementation of a Food is Medicine Prescription program.
